# O.5.1-5 Influence of the parental perception of the neighborhood environment on physical activity and active school commuting in Ecuadorian children

**DOI:** 10.1093/eurpub/ckad133.235

**Published:** 2023-09-11

**Authors:** Samuel Adrián Escandón, María José Molina, Vinicio Sanchez, Angélica Ochoa, Delfien Van Dyck

**Affiliations:** Universidad de Cuenca, Ecuador; Universidad de Cuenca, Ecuador; Universidad Politécnica Salesiana Sede Cuenca; Universidad de Cuenca, Ecuador; Ghent University; angelica.ochoa@ucuenca.edu.ec

## Abstract

**Purpose:**

Physical inactivity among children is considered a multidimensional problem. This study examines the relationships between parents' perceptions of the neighborhood environment with physical activity (PA) and active school commuting among urban children from Cuenca-Ecuador.

**Methods:**

A cross-sectional study was conducted between October 2018 and March 2019 among 1028 children (8 to 12 years old) and their parents/guardians. Children's PA levels were objectively measured using accelerometers (Actigraph). Parent's perceptions of their neighborhood aesthetics, traffic, and crime hazards were assessed using the Neighborhood Environment Walkability Scale for Youth (NEWS-Y) parent version. Active school commuting (ASC) was evaluated using a validated tool applied to the children. Children who reported walking or using a bicycle to commute to and from school five days a week were classified as active commuters. Multivariate ordinary least squares and logistic regressions models were performed to determine associations between parents' perceptions of the neighborhood environment with PA and ASC, respectively.

**Results:**

Complete data were available from 711 children (52% female) and 521 parents. The parental perceptions of the neighborhood aesthetics (Mean: 2.68; DE:0.75) and traffic hazards (Mean: 2.44; DE:0.44) were not associated with children's physical activity. Neighborhood crime safety (Mean:2.64; DE:0.74) score was negatively associated with the children's vigorous physical activity (Mean:14.50 minutes/day; DE:8.66) level (β = -1.52 minutes/day; DE = 0.52; p < 0.004). After adjusting for walking distance and car ownership, parental perception of neighborhood aesthetics, traffic, and crime hazards were not associated with ASC.

**Conclusions:**

Crime hazard was identified as the main barrier to children's vigorous PA. The neighborhood environment seems not to be associated with children's ASC. Future studies should identify the determinants for ASC, and comprehensive policies are urgently needed to overcome crime hazards in Ecuador

**Funding source:**

Universidad de Cuenca, Universidad Politécnica Salesiana Sede Cuenca.

